# DOES IT WORK? -a randomized controlled trial to test the efficacy of HCV and HIV-related education on drug users in MMT, China

**DOI:** 10.1186/s12879-019-4421-5

**Published:** 2019-09-05

**Authors:** Jing Ying Zhang, Zhi Bin Li, Lei Zhang, Jun Wang, Le Ping Huang, Gui Lai Zhan, Zhu Li, Jiang Du, Min Zhao

**Affiliations:** 1 Collaborative Innovation Center for Brain Science, Shanghai Mental Health Center, Shanghai Jiao Tong University School of Medicine, 600 South Wanping Road, Shanghai, 200030 China; 2 Mental Health Center of Jiading District in Shanghai, Shanghai, China; 3 Mental Health Center of Yangpu District in Shanghai, Shanghai, China; 4 Mental Health Center of Hongkou District in Shanghai, Shanghai, China; 5 Mental Health Center of Xuhui District in Shanghai, Shanghai, China; 6 Songnan Community Health Service Center, Baoshan District, Shanghai, China; 7 Shanghai Key Laboratory of Psychotic Disorders, Shanghai, China

**Keywords:** HCV, HIV, Drug users, MMT, Knowledge, Infection awareness

## Abstract

**Background:**

HCV (Hepatitis C virus) is a prevalent chronic disease with potentially deadly consequences, especially for drug users. However, there are no special HCV or HIV (human immunodeficiency virus)-related intervention programs that are tailored for drug users in China; to fill this gap, the purpose of this study was to explore HCV and HIV-related knowledge among drug users in MMT (methadone maintenance treatment) sites of China and to investigate the effectiveness of HCV and HIV-related education for improving the knowledge of IDUs (injection drug users) and their awareness of infection.

**Methods:**

The study was a randomized cluster controlled trial that compared a usual care group to a usual care plus HCV/HIV-REP (HCV/HIV-Reduction Education Program) group with a 24-week follow-up. The self-designed questionnaires, the HCV- and HIV-related knowledge questionnaire and the HIV/HCV infection awareness questionnaire, were used to collect the data. Four MMT clinics were selected for this project; two MMT clinics were randomly assigned to the research group, with subjects receiving their usual care plus HCV/HIV-REP, and the remaining two MMT clinics were the control group, with subjects receiving their usual care over 12 weeks. Sixty patients were recruited from each MMT clinic. A total of 240 patients were recruited. Follow-up studies were conducted at the end of the 12th week and the 24th week after the intervention.

**Results:**

At baseline, the mean score (out of 20 possible correct answers) for HCV knowledge among the patients in the group receiving the intervention was 6.51 (SD = 3.5), and it was 20.57 (SD = 6.54) for HIV knowledge (out of 45 correct answers) and 8.35 (SD = 2.8) for HIV/HCV infection awareness (out of 20 correct answers). At the 12-week and 24-week follow-up assessments, the research group showed a greater increase in HCV−/HIV-related knowledge (group × time effect, F = 37.444/11.281, *P* < 0.05) but no difference in their HIV/HCV infection awareness (group × time effect, F = 2.056, *P* > 0.05).

**Conclusion:**

An MMT-based HCV/HIV intervention program could be used to improve patient knowledge of HCV and HIV prevention, but more effort should be devoted to HIV/HCV infection awareness.

**Trial registration:**

Protocols for this study were approved by institution review board (IRB) of Shanghai Mental Health Center (IRB:2009036), and registered in U.S national institutes of health (http://www.clinicaltrials.gov, NCT01647191). Registered 23 July 2012.

## Background

HCV is a prevalent chronic disease with potentially deadly consequences. The global HCV positive prevalence is forecasted at 1.1% (0.9–1.4%), with an estimated population of 80 (64–103) million patients [1]. China also faces a similar situation, with an HCV prevalence among the general Chinese population varying from 0.43 to 2.2%, corresponding to a range of 6 million to 30 million people [2, 3]. High risk factors for HCV infection include injection drug use and transfusion of blood products [4]. Since a security system was established for blood donors in China, IDU has been the predominant mode of HCV transmission. According to the 2017 Annual Report on Drug Control in China, approximately 2.50 million drug users were documented in China at the end of 2016, but the actual number is estimated to be approximately 14 million [5]. Thus, it is not surprising that HCV infection prevalence among IDU in China is thought to be very high, ranging from 15.6 to 98.7% in different provinces [6].

Chronic HCV infection is associated with substantial morbidity and mortality. It is a major cause of liver and liver-related death, and HCV has surpassed HIV as a cause of death in the US [7, 8]. Globally, the burden of HCV infection is expected to substantially increase within the next few decades [9]. The development of chronic HCV infection may lead to progressive hepatic fibrosis, cirrhosis, and carcinoma [10]. However, most individuals who are infected with HCV are unaware of their infection because HCV can be asymptomatic for decades. Despite the serious consequences of HCV infection among IDUs, treatment uptake remains rather low, even in countries where the treatment is available and affordable [11]. A growing body of research has indicated that the barriers to treatment for HCV-positive drug users include limited knowledge, low risk awareness among patients and health service providers, discrimination of IDUs, and the high price of medication [12, 13]. Although the barriers for accessing treatment have been described, there is still a lack of evidence-based research to help guide future policy and treatment plans for drug users, especially in Asian countries. For example, even though in 2002 the U.S. National Institutes of Health (NIH) Hepatitis C Consensus Conference recommended treatment for HCV for IDUs to be determined on a case-by-case basis [14], this recommendation has not been followed by China. According to the Hepatitis C Prevention Guidelines in China [15], active drug users are excluded from antiviral treatment. Given the current situation in China and the high relapse rate among drug users, it is necessary to scale up education efforts to improve knowledge about HCV to help slow the progression of HCV in patients or to avoid infection in HCV-negative drug users.

A drug abuse treatment program has been shown to be a good platform to deliver HCV and HIV-related intervention or medical services. As a strategy for solving the problems of opiate abuse and HIV, the Chinese government established MMT clinics throughout the country starting in 2004. By the end of 2016, there were more than 760 MMT clinics in the country, and 300,000 heroin users benefit from this service [16]. MMT clinics have been shown to be an effective method to prevent the spread of HIV/AIDS [17, 18]. Given their same route of transmission and the situation in China, incorporating HCV prevention/intervention strategies into the MMT setting could be done more fully and effectively to prevent HCV-related complications and increase medical uptake.

At present, there are no special HCV and HIV-related intervention programs that have been tailored for drug users in China; to fill this gap, this cluster-randomized study was designed to explore the HCV- and HIV-related knowledge among drug users in MMT sites and to investigate whether an HCV and HIV intervention program was effective in improving their knowledge and infection awareness.

## Methods

### Study design

The study was a randomized cluster design with the MMT clinic as the unit of randomization to evaluate the efficacy of the intervention. Interested and eligible participant volunteers were invited into either the usual care group or the usual care plus HCV/HIV-REP (HCV/HIV-Reduction Education Program) group, depending on the randomization results. Four MMT clinics with similar demographic characteristics were selected for this study – two clinics for HCV/HIV-REP and two clinics for the usual care sites.

Usual care sites: Those in the usual care sites received standard procedures at the MMT for 12 weeks and were required to participate in follow-up interviews at the end of 12 weeks and 24 weeks after the intervention. The current standard for the usual care at an MMT includes a physical exam (including HIV testing, HCV testing, and urine testing for opiates) and weekly consultation. Patients were expected to take daily methadone under supervision.

HCV/HIV-REP sites: In addition to usual care services, the subjects in the HCV/HIV-REP sites received twelve 1.5-h sessions over 12 weeks based on the education materials that are described below.

Protocols for this study were approved by institution review board (IRB) of Shanghai Mental Health Center (IRB:2009036), and registered in U.S national institutes of health (http://www.clinicaltrials.gov, NCT01647191).

### Participants and recruit procedures

The criteria of MMT clinics are as follows: (1) a minimum of 100 MMT patients in the clinic; (2) adequate space to accommodate the research assistants and study protocol procedures, including focus group discussions; (3) able to provide complete data on individual patients regarding their attendance and their HAV, HBV, HCV, HIV and other lab test results, which will be shared upon the patient’s consent.

In total, there are thirteen MMT clinics in Shanghai, and the research assistant contacted the directors of each MMT clinics via a call and explained the research contents to them. Eight MMT clinics meet the criteria mentioned above, and two of them expressed that they were not interested in attending this program. For convenience, we the excluded Baoshan and Jinshan MMTs because they are far from the research institutes. Finally, four MMT clinics were selected for this project (Xuhui MMT clinic, Minhang MMT clinic, Yangpu MMT clinic, and Hongkou MMT clinic); two MMTs were randomly assigned to the research group (HCV/HIV-REP sites) receiving standard methadone maintenance treatment plus HCV intervention for 12 weeks, and the remaining two MMT clinics were the control group (usual care sites) receiving standard methadone maintenance treatment.

Patients were recruited by posting advertisements at the MMT clinics, and the eligibility criteria included: (1) aged 18–65 years; (2) met DSM-IV (Diagnostic and Statistical Manual of Mental Disorders-IV) criteria for heroin dependence; (3) consented to join this study and only participate this study; (4) signed informed consent. Individuals were excluded if they had any mental illness or organic disease that would prevent them from completing the survey or if they failed to attend therapy.

**Fig. 1:**
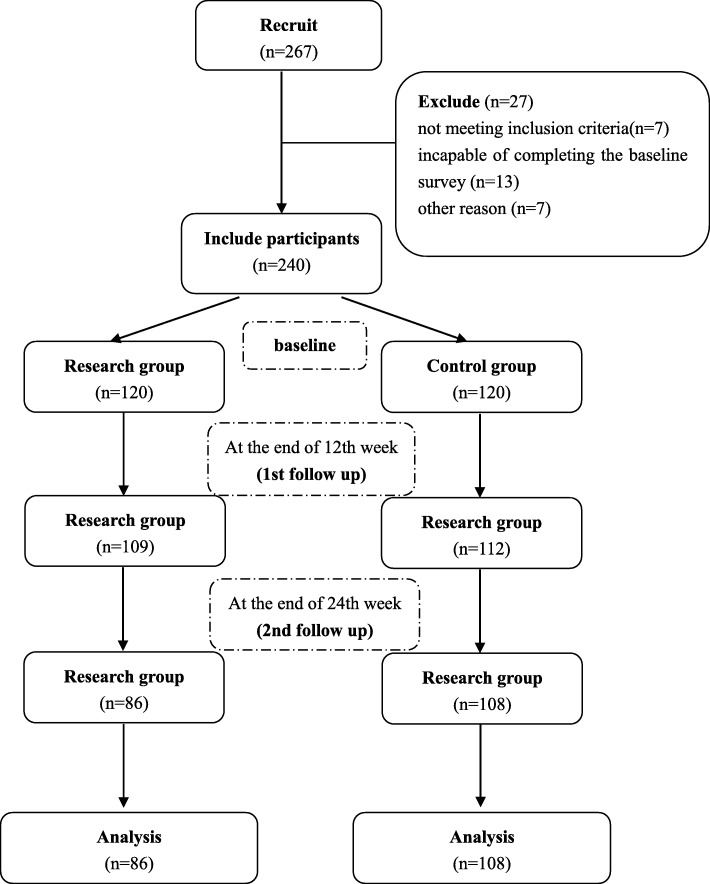
The research flowchart. A total of 240 patients were included in this study. A total of 221 patients and 194 patients attended the follow-up interview at the end of the 12th week and 24th week after the intervention

A total of 267 patients were invited to attend the screening interview, and 27 of them were excluded because they did not meet the inclusion criteria (*n* = 7), were incapable of completing the baseline survey (*n* = 13), or for other reasons (n = 7). A total of 221 patients and 194 patients attended the follow-up interview at the end of the 12th week and 24th week after the intervention (Fig. 1 flowchart).

### Intervention

#### Develop the HCV/HIV-REP (HCV/HIV-reduction education program) protocol

Before the intervention, experts in both the addiction domain and the HCV domain were invited to discuss the contents of the intervention. Doctors and patients from MMT clinics were also consulted through focus interviews to understand their requirements. After the information collecting period, the principle investigator (PI) and research assistants made a draft of the intervention protocol based on the information that was collected from the focus group and experts. Five patients and five staff members were invited from the MMT clinics to pilot test the draft, to ensure they understand the contents of the protocol and allow them to provide suggestions or advice for the protocol. Finally, the research staff modified and finalized the drafts based on the suggestions from experts, MMT staff members, and patients.

#### Procedure

The HCV/HIV-REP was provided in the form of a group intervention. Approximately 15–20 patients were included in each session, which lasted 1.5 h. In the first 15 min, the participants were asked to recapitulate their last session, and in the last 15 min they were led to summarize the current day’s session. Another hour consisted of delivering education by a variety of learning techniques, including lectures, brainstorming, small and large group activities, individual worksheets, role-playing, and playing videos. For example, small teams of up to 5 participants conducted role-plays; large groups were assembled to encourage discussion about HCV/HIV-related risk reduction behaviors. Patients were encouraged to discuss issues related to each session’s topic. Pre- and posttraining tests were conducted via hard copy questionnaires at each session. The posttraining was conducted immediately after each session to evaluate the patients’ comprehension of the session. All of the educational programs were delivered by the PI and research assistant, who had extensive knowledge on drug abuse and the HCV/HIV education program. Each patient received RMB30 or its equivalent for compensation. In total, each participant in the research group received 12 interventions.

***The contents of the education:***

1. The liver and its function: Explain the anatomy of the liver and its function.
2. Types of hepatitis: Explain the types of hepatitis and the relationships of the different types.
3. HCV/HIV knowledge: Provide HCV/HIV-related knowledge about transmission routes, how to test for HCV/HIV, the genotype of HCV, and the possible outcomes of HCV infection.
4. Alcohol and HCV: Clarify how alcohol affects the risk of developing liver cancer for HCV-positive drug users.
5. HCV/HIV and risk behavior: Explain the risk behaviors that are related to HCV/HIV infection, including sharing needles and unprotected sexual behaviors and how to prevent risk behaviors during daily life.
6. HCV/HIV treatment: provide current treatment information for HCV/HIV infection, and how to deal with side effect.
7. HCV/HIV prevention and intervention: How to prevent HCV/HIV infection for HCV/HIV-negative drug users and how to slow the progression of disease.
8. HIV/HCV coinfection: Introduce the natural history of HIV/HCV coinfection and the impact of HIV on HCV infected patients.
9. Summary: Summarize the former topics, encourage the patients to discuss what they had learned, and address their questions.

### Materials

#### The self-designed questionnaire

The demographic characteristics, including age, gender, education, marriage, and drug (alcohol) use history of each patient were collected via a self-designed questionnaire.

#### HCV-related knowledge questionnaire

This questionnaire was developed by the National Development and Research Institutes (NDRI) to assess the HCV related knowledge and attitude of the addicts and staffs with 20 items. The answer was “Yes”, “No” or “I don’t know,” and the score of the HCV-related knowledge was defined as the total number of correct responses. Specifically, “I don’t know” was defined as wrong. The instrument was translated into Chinese by one psychiatrist and back-translated by another psychiatrist and has been used in patients with a Chinese background. [19]

#### HIV related knowledge questionnaire

HIV knowledge were evaluated using a 45 items HIV Knowledge Questionnaires (HIV-K-Q) developed by Michael and his colleges in 1997 [20]. One point is given for each correct answer with a possible score ranging from 0 to 45.

#### HCV/HIV infection awareness

This is a self-rating questionnaire containing four questions: it’s impossible for me to be infected with HIV/HCV; it’s possible for me to be infected HIV/HCV; I might have sex with HIV/HCV-infected people; and maybe I have been infected with HIV/HCV. Each item is scored on a 1–5 Likert scale from “1 strongly disagree” to “5 strongly agree” with cumulative scores ranging from 0 to 20. The score of question 1 was reversed when the total scores were calculated.

#### Additional data to be collected

Research staff will obtain from clinic records information about HIV, HAV, HBV, and HCV antibody test results, treatment attendance, and other information relevant to this study for each participating subject.

### Data analysis

Statistical Product and Service Solutions 20.0 (SPSS 20.0) was used to conduct statistical comparisons between control and intervention groups. The significance level was set at 0.05. A t test and a chi-square test were conducted to compare the demographic characteristics and the drug (alcohol) use history between the two clusters, investigating the success of the randomization. Independent t tests were used to compare the distribution of the continuous variables, and chi-square tests were used to compare the distribution of the categorical variables. The effectiveness of HCV-REP intervention on HIV/HCV knowledge and awareness of HIV/HCV infection were examined using linear mixed models. We included group (research and control), time (baseline, 12th week and 24th week), and time×group interaction, with the time×group interaction term indicating a differential change by group from baseline to the end of the trial. We also included the MMT clinics as random variables to include the cluster level effect.

## Results

### Baseline assessments and drug use history

A total of 267 MMT patients were recruited from the four selected MMT clinics, with 27 (10.1%) excluded due to different reasons (Fig. 1). A total of 19 subjects did not complete the 12-week follow-up assessment, and 46 subjects did not complete the 24-week follow-up assessment. There were no significant differences between those who did and did not complete the 12-week and 24-week follow-up interviews in terms of their age, gender, education, marriage, and length of receiving the methadone maintenance treatment.

A total of 240 MMT patients were recruited, and the average age was 42.46 (SD = 8.6); 192 (80%) patients were male, and 108 (45%) had medical insurance. The average duration of heroin use was 10.89 (SD = 6.42) years, and the average time they received MMT was 2.48 (SD = 1.36) years. A total of 80 (37.08%) patients used alcohol. In terms of HCV/HIV infection, 70% of patients (168) were HCV positive based on their medical records, and no participants were HIV positive. The average scores of HCV- and HIV-related knowledge was 6.51 (SD = 3.5) and 20.57 (SD = 6.54), respectively. There were no significant differences in the HCV/HIV infection or knowledge scores between the two groups, but we did observe significant differences in alcohol use and HIV/HCV infection awareness (Table 1).

**Table 1:** Baseline characteristics of participants (* *p* < 0.05, **p < 0.001)

	Research	Control	Total	*p*
	(*n* = 120)	(n = 120)	(*n* = 240)	
Age, M(SD), years	43.15(8.21)	41.83(8.93)	42.46 (8.6)	0.260
Male, n (%)	98 (81.67)	94(78.33)	192(80.0)	0.630
Marital status, n (%)				
Single	63(52.5)	62(51.7)	125(52.08)	0.788
Married	57(47.5)	58(48.3)	115(47.92)	
Education, M(SD), years	9.84(1.87)	9.94(2.28)	9.89(2.08)	0.710
No employment, n (%)	91(75.84)	87(72.5)	178(74.2)	0.550
Have medical insurance, n (%)	60(50.0)	48(40.0)	108(45.0)	0.120
HCV infection, n (%)	85(70.83)	83(69.16)	168(70)	0.358
HIV infection, n (%)	0	0	0	–
duration of heroin use, M(SD), years	11.51(7.97)	10.40(5.13)	10.89(6.42)	0.203
length of receiving MMT, M(SD), years	2.37(1.24)	2.68(1.45)	2.48(1.36)	0.410
With alcohol use, n (%)	28(23.3)	52(43.3)	80(37.08)	0.001**
HCV related knowledge, M(SD)	6.923(3.165)	6.10(3.77)	6.51(3.5)	0.072
HIV related knowledge, M(SD)	21.30(6.91)	20.14(9.73)	20.57(6.54)	0.695
HCV/HIV infection awareness	7.89(2.31)	9.06(2.99)	8.35(2.8)	0.001**

### Assessments at the 12-week and 24-week follow-up visits

1. HCV knowledge: The linear mixed models analysis revealed the main effects of group (F = 107.282, *p* < 0.001) and time (F = 77.672, p < 0.001), and a significant group x time interaction (F = 37.444, p < 0.001). The research group showed a greater increase in HCV knowledge.
2. HIV knowledge: The linear mixed models analysis revealed the main effects of group (F = 37.633, p < 0.001) and time (F = 15.441, p < 0.001), and a significant group x time interaction (F = 11.281, p < 0.001). Compared with the control group, the research group showed a greater increase in HIV knowledge.
3. HCV/HIV infection awareness: The linear mixed models analysis revealed the main effect of group (F = 13.496, p < 0.001), but no time effect (F = 0.246, *p* = 0.782) or group x time interaction (F = 2.056, *p* = 0.086) (Table 2).

**Table 2:** The effectiveness of intervention on HCV/HIV knowledge and HCV/HIV infection awareness (* *p* < 0.05, **p < 0.001)

		Research	Control	Group (F, *p*)	Time (F, p)	Group × Time (F, *p*)
		*n* = 86 (mean, SD)	*n* = 108 (mean, SD)			
HCV knowledge	Baseline	6.73(3.180)	5.94(3.745)	107.282 0.000**	77.672 0.000**	37.444 0.000**
	12-week	12.55(3.465)	7.03(3.718)			
	24-week	12.25(3.105)	8.36(3.028)			
HIV knowledge	Baseline	21.05(7.026)	18.71(9.434)	37.633 0.000**	15.441 0.000**	11.281 0.000**
	12-week	24.83(6.092)	18.83(9.584)			
	24-week	25.87(5.873)	21.24(6.237)			
HCV/HIV infection awareness	Baseline	9.47(1.352)	10.28(2.419)	13.496 0.000**	0.246 0.782	2.056 0.086
	12-week	9.41(1.758)	10.25(2.478)			
	24-week	9.63(1.776)	9.76(1.727)

## Discussion

The HCV epidemic is an important problem that has been coined a viral time bomb. People who use injected drugs are heavily affected by this infectious disease. However, despite its high prevalence, HCV testing, prevention, assessment, and treatment in drug users remain suboptimal. A body of research has demonstrated that HCV education can reduce risk behaviors and disparities in HCV infection and influence a patient’s decision to explore and initiate antiviral treatment [21–24]. As the first randomized study among drug users in China, the study indicated that the high prevalence of HCV (70%) among drug users in MMTs in Shanghai was higher than the prevalence estimates of 67.0% from the meta-analyses among IDUs and 60.1% among IDUs in MMTs in China [25]; it was also higher than the global HCV prevalence among drugs users (67%) [26]. While the intervention outcomes are in line with our expectations that the knowledge of HIV and HCV increased, there were no changes in infection awareness after the intervention.

A body of research has indicated that behavior change is a complicated process that can be affected by many factors. According to the information–motivation–behavioral (IMB) skills model proposed by Fisher to explain the process of behavioral change, information or knowledge is defined as a ‘prerequisite’ for enacting a health behavior among three constructs (information or knowledge, motivation and behavioral skills) [27]. This means that, even though there were no actual behavior changes after the intervention, the improvements in HCV/HIV knowledge and willingness to change would act as positive factors to initiate the process of behavior changes in the patients.

At present, there is no vaccine that is currently available to prevent HCV infection. However, HCV is a preventable disease, especially for drug users. WHO guidance has indicated that the basic requirements for successful HCV prevention should provide drug users access to health care and justice, health literacy, and adapted services, and the key measurements for effective HCV prevention include needle exchange programs and opioid substitution programs [28]. In China, to solve the problems of HIV infection among IDUs, the government established the MMT program in 2004, but it does not appear to work for HCV prevention, based on the data of the HCV infection rate among MMT programs from 2004 to 2012 [25, 29]. Most researchers have pointed out that the combination of these two preventive steps at a high coverage could minimize the risk of HCV seroconversion by up to 75–80% [30, 31], but in China, only approximately 2% of IDUs have been able to access needle exchange programs [32], which may weaken the efficacy of MMTs on HCV infection prevention. Therefore, it is necessary to expand needle exchange programs to supplement MMTs in China.

In the current study, the HCV infection rate was 70%, which means that medical treatment or related counseling for this group was needed. Researchers have suggested that it would be beneficial for HCV positive clients to receive treatment for HCV at their MMT program [33, 34], but cost is a significant barrier to them because both HCV treatment and MMT programs are not free in China. Given the poor living conditions of drug users, it is almost impossible for them to afford this medical expense. Moreover, active drug users are excluded from antiviral treatment, according to the Hepatitis C Prevention guidelines in China [35]; this exclusion provides another barrier from the government level and explains why, even though medication to treat the virus is available, many drug users have been unable to reap the benefits of HCV treatment. Other barriers include limited knowledge, lack of empirical data in the current study, and being unable to confirm the barriers and problems in accessing HCV-related treatment in this group. Under this condition, providing education and improving the knowledge level should be an appropriate strategy to slow the progression of HCV infection.

We acknowledge a number of limitations in the study: first, since all of the participants were recruited from MMT clinics, dissemination of the results should be done with caution. Future studies may involve a randomization study in other treatment sites or areas. Second, a quantitative study cannot define the barriers for accessing HCV treatment in this group, so a future study should combine quantitative and qualitative research. Finally, due to self- reported data, the recall bias could be as a confounding factor for the outcomes.

## Conclusion

An MMT-based HCV/HIV intervention program could be used to improve patient knowledge of HCV and HIV prevention, but more effort will need to be devoted to HIV/HCV infection awareness. Overall, this pilot study confirmed the effects of HCV- and HIV-related intervention among drug users in MMT programs in China, which have been defined as situations for delivering HCV/HIV intervention; although there are limitations in the current study, it still provided evidence-based support for China and other Asian countries for how to deliver HCV- and HIV-related interventions based on the MMT programs.

## Data Availability

The datasets generated and analyzed during the current study are not publicly available due to the conflict with patients’ privacy (it was not in accordance with patients’ written informed consent) but are available from the corresponding author on reasonable request.

## Abbreviations

HCV: hepatitis C virus
HCV/HIV-REP: HCV/HIV-reduction education program
HIV: human immunodeficiency virus
IDU: injection drug user
IRB: institution review board
MMT: methadone maintenance treatment
NIH: the U.S national institutes of health
US: the United States
